# Sirt1 sustains female fertility by slowing age‐related decline in oocyte quality required for post‐fertilization embryo development

**DOI:** 10.1111/acel.13204

**Published:** 2020-07-30

**Authors:** Juvita D. Iljas, Zhe Wei, Hayden A. Homer

**Affiliations:** ^1^ The Christopher Chen Oocyte Biology Research Laboratory Centre for Clinical Research The University of Queensland Herston Qld Australia

**Keywords:** ageing, fertility, mitochondria, oocyte, oxidative stress, Sirt1

## Abstract

The NAD^+^‐dependent sirtuin deacetylase, Sirt1, regulates key transcription factors strongly implicated in ageing and lifespan. Due to potential confounding effects secondary to loss of Sirt1 function from the soma in existing whole‐animal mutants, the in vivo role of Sirt1 in oocytes (oocyte‐Sirt1) for female fertility remains unknown. We deleted Sirt1 specifically in growing oocytes and study how loss of oocyte‐Sirt1 affects a comprehensive range of female reproductive parameters including ovarian follicular reservoir, oocyte maturation, oocyte mitochondrial abundance, oxidative stress, fertilization, embryo development and fertility during ageing. Surprisingly, eliminating this key sirtuin from growing oocytes has no effect in young females. During a 10‐month‐long breeding trial, however, we find that 50% of females lacking oocyte‐Sirt1 become prematurely sterile between 9 and 11 months of age when 100% of wild‐type females remain fertile. This is not due to an accelerated age‐related decline in oocyte numbers in the absence of oocyte‐Sirt1 but to reduced oocyte developmental competence or quality. Compromised oocyte quality does not impact in vivo oocyte maturation or fertilization but leads to increased oxidative stress in preimplantation embryos that inhibits cleavage divisions. Our data suggest that defects emerge in aged females lacking oocyte‐Sirt1 due to concurrent age‐related changes such as reduced NAD^+^ and sirtuin expression levels, which compromise compensatory mechanisms that can cover for Sirt1 loss in younger oocytes. In contrast to evidence that increasing Sirt1 activity delays ageing, our data provide some of the only in vivo evidence that loss of Sirt1 induces premature ageing.

## Graphical abstract

The very first oocyte‐specific Sirt1‐knockout mouse was generated in this study. Mice lacking Sirt1 activity in their oocytes were phenotypically indistinguishable whilst young, but became prematurely sterile between 9 and 11 months of age. The latter was not due to decline in oocyte numbers, but rather due to decline in oocyte quality. Here, we discovered that Sirt1 plays a critical role in slowing age‐related decline in oocyte quality required for post‐fertilization embryogenesis.

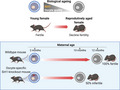

## 1 INTRODUCTION

Mammalian oocytes are heavily reliant on mitochondrial oxidative phosphorylation (OXPHOS) for ATP generation using pyruvate derived from surrounding cumulus cells (Eppig, 1976; Johnson, Freeman, Gardner, & Hunt, 2007). Notably, mitochondria produce the vast majority of cellular free radicals and reactive oxygen species (ROS), which arise as a by‐product of OXPHOS (Figueira et al., 2013; May‐Panloup et al., 2016; Verdin, Hirschey, Finley, & Haigis, 2010). ROS performs important signalling functions but is also highly reactive and damaging to fundamental cellular components such as DNA and proteins. Mitochondrial de‐regulation and increased oxidative stress are therefore thought to be major drivers underpinning age‐related decline in oocyte developmental competence (or quality) (May‐Panloup et al., 2016).

Sirtuins are a family of NAD^+^‐dependent deacetylases/ADP‐ribosyl transferases/deacylases important for a plethora of functions (Haigis & Sinclair, 2010). Of the seven sirtuins (Sirt1‐7) found in mammals, Sirt1 is the most extensively studied, is primarily nuclear in location and regulates key transcription factors (Haigis & Sinclair, 2010; Tang, 2016). Included amongst these are tumour suppressors regulating cell death and survival such as p53 (Vaziri et al., 2001) and Forkhead box (Foxo) family members (Brunet et al., 2004) as well as PPARγ coactivator‐1α (PGC‐1α) (Brunet et al., 2004; Gerhart‐Hines et al., 2007; Lagouge et al., 2006), a master regulator of mitochondrial biogenesis. By controlling nuclear respiratory factor (NRF) 1 and 2, PGC‐1α indirectly regulates mitochondrial transcription factor A (TFAM), which stimulates mitochondrial DNA replication and mitochondrial gene expression (Tang, 2016). Through its effects on Foxo3a, which regulates the expression of the antioxidant manganese superoxide dismutase (MnSOD) (Kops et al., 2002), Sirt1 also has important antioxidant functions. Collectively therefore, by modulating cell survival, mitochondrial integrity and antioxidant defence, Sirt1 is strongly linked to ageing and lifespan.

The in vivo role of Sirt1 in oocytes and the importance of oocyte‐Sirt1 for female fertility are unclear. Whole‐body Sirt1‐null mice (Sirt1^−/−^) and mice deleted of Sirt1 exon 4 (Sirt1^ΔEx4/ΔEx4^) are frail with multiple developmental defects and increased postnatal mortality (Cheng et al., 2003; McBurney et al., 2003). Sirt1^−/−^ females that do survive to adulthood fail to produce litters when paired with wild‐type males (McBurney et al., 2003). However, this is not due to a lack of developmentally competent oocytes but results from a defect in hypothalamic‐pituitary hormones responsible for driving follicular development (Kolthur‐Seetharam et al., 2009). Consequently, exogenous hormonal stimulation of Sirt1^−/−^ females produces normal numbers of mature eggs, which generate viable pups following in vitro fertilization (IVF) (Coussens, Maresh, Yanagimachi, Maeda, & Allsopp, 2008). Mutant mice harbouring a point mutation within the Sirt1 catalytic domain (H355Y) that eliminates Sirt1 catalytic activity (Sirt1^Y/Y^) have lower postnatal mortality than Sirt1^−/−^ animals (Seifert et al., 2012). Unlike Sirt1^−/−^ females, homozygosity for the H355Y mutation did not affect female fertility, at least not whilst young (Seifert et al., 2012). Because of potential confounding effects brought about by the loss of Sirt1 from other cell types and the resulting compromise to overall fitness, the in vivo role of oocyte‐Sirt1 in female fertility, and the effects of stressors such as ageing, cannot adequately be studied using whole‐body mutants. Increasing Sirt1 activity using either Sirt1 activators (resveratrol or SRT1720) (Liu et al., 2013; Zhou et al., 2014), transgenic Sirt1 over‐expression in oocytes (Long et al., 2019) or by calorie restriction (Liu et al., 2015) sustains the ovarian follicular pool and/or oocyte quality during ageing. Thus, increasing Sirt1 function appears to combat deleterious effects of reproductive ageing in vivo but whether Sirt1 loss causes premature deterioration is unknown.

Here, we generate oocyte‐specific Sirt1‐knockout mice for the first time. We find that loss of Sirt1 in oocytes has no impact on ovarian reserve, oocyte chromosome segregation, mitochondrial function, antioxidant defence or litter sizes whilst young. However, with ageing, livebirth rates decline more steeply when oocytes lack Sirt1. This is not because of reduced oocyte numbers but due to reduced quality. Although aged Sirt1‐deficient oocytes contain less mitochondria, this does not impact oocyte ATP levels, maturation or in vivo fertilization. Instead, our data suggest that post‐fertilization events become impaired due to increased oxidative stress in embryos that compromises early cleavage divisions. Highly significantly, reducing embryonic ROS using the antioxidant *N*‐acetyl cysteine (NAC) rescues embryonic division. Our data suggest that maternally derived Sirt1 becomes increasingly critical for embryonic development during ageing due to the concurrent decline in overall sirtuin function, which undermine compensatory mechanisms.

## 2 RESULTS

### 2.1 Ovarian reserve is unaffected by oocyte‐specific loss of Sirt1 deacetylase activity

We deleted exon 4 of *Sirt1* encoding 51 amino acids of the conserved catalytic domain (Figure S1a) specifically in oocytes. To generate mice lacking Sirt1 only in oocytes, we crossed females in which, exon 4 is flanked by *loxP* sites (*Sirt1^f^* ^/^ *^f^*) (Cheng et al., 2003) with *Zp3*‐*Cre* males (Figure S1b). Since Cre recombinase expression is driven by the *Zp3* promoter, which is only expressed in growing oocytes (Lewandoski, Wassarman, & Martin, 1997), exon 4 deletion in *Zp3*‐*Cre*;* Sirt1^f^* ^/^ *^f^* mice is restricted to oocytes. Thus, *Zp3*‐*Cre*;* Sirt1^f^* ^/^ *^f^* oocytes contain truncated *Sirt1* lacking exon 4 (*Sirt1^ΔEx4^*) whereas ear clippings retain full‐length *Sirt1^f^* ^/^ *^f^* (Figure S1c). In line with this, oocytes from *Zp3*‐*Cre*;* Sirt1^f^* ^/^ *^f^* mice (hereafter referred to as OoSirt1^ΔEx4/ΔEx4^ mice) expressed a truncated protein exhibiting increased mobility during immunoblotting compared with oocytes from *Sirt1^f^* ^/^ *^f^* mice (hereafter referred to as OoSirt1^+/+^ mice) (Figure S1d).

In mice, oocytes undergo a 2–3 week growth phase during which time, follicles develop from primordial to primary, secondary and, finally, antral stages. Since increasing Sirt1 activity sustains a higher follicular pool during ageing (Liu et al., 2013; Long et al., 2019; Zhou et al., 2014), we reasoned that loss of Sirt1 might be detrimental to follicular development. Surprisingly, however, we found no difference in numbers of primordial, primary, secondary or antral follicles between young OoSirt1^ΔEx4/ΔEx4^ mice and OoSirt1^+/+^ mice (Figure S2a,b). In keeping with similar numbers of antral stage follicles, we also obtained similar numbers of fully grown germinal vesicle (GV)‐stage oocytes from young OoSirt1^ΔEx4/ΔEx4^ mice and OoSirt1^+/+^ mice following hormonal stimulation (Figure S2c). Thus, loss of Sirt1 catalytic activity during oocyte growth does not affect follicle development or ovarian reserve in young mice.

### 2.2 Lack of Sirt1 does not affect antioxidant defence, mitochondrial function or in vivo fertility in young females

**FIGURE 1:**
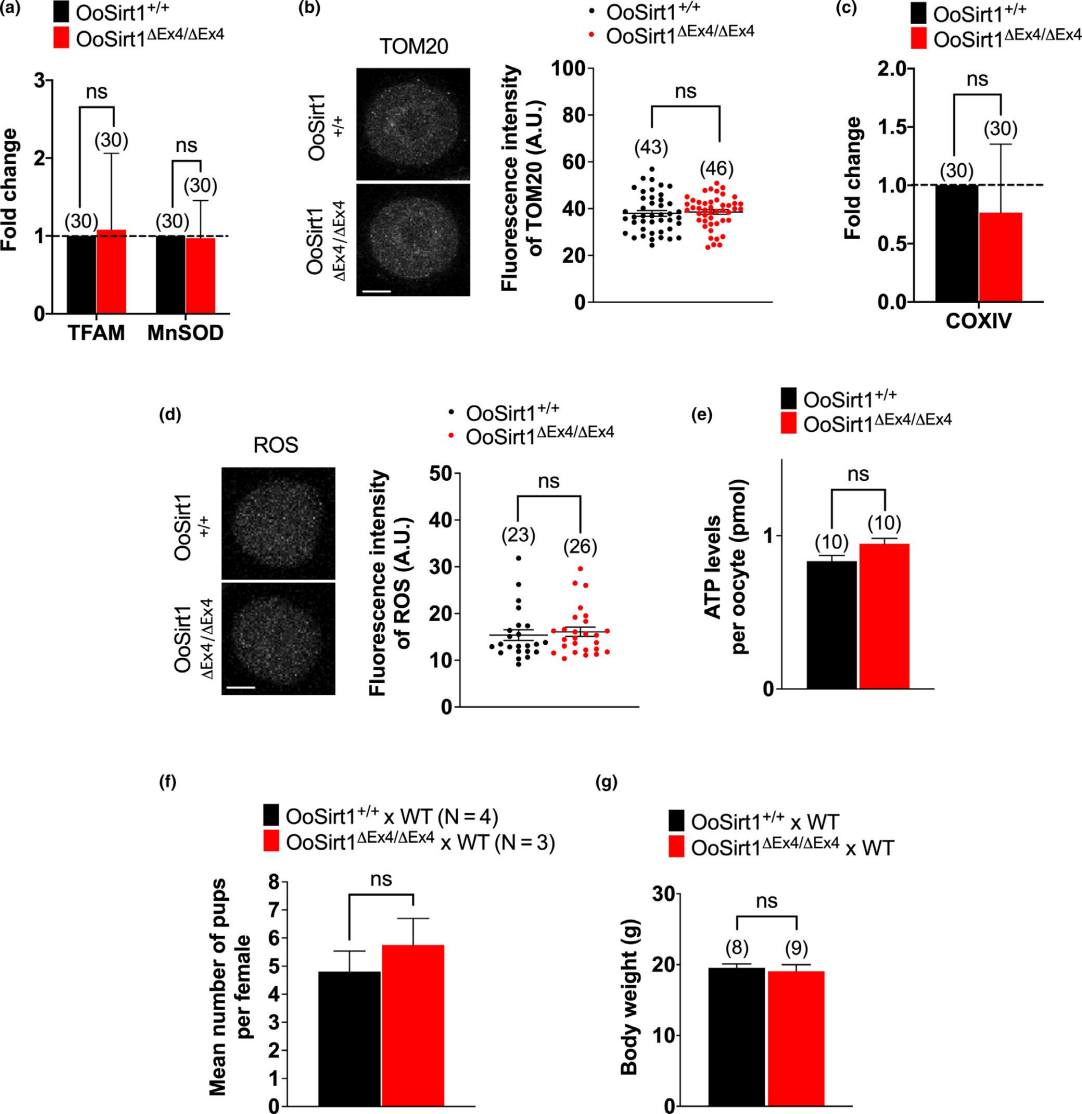
Mitochondrial abundance, oxidative stress and ATP levels in oocytes and in vivo fertility in young females. (a–e) Oocyte levels of *TFAM* and *MnSOD* mRNA (a), TOM20 (b), *COXIV* mRNA (c), ROS (d) and ATP (e). Panels show images of representative OoSirt1^ΔEx4/ΔEx4^ and OoSirt1^+/+^ oocytes stained for TOM20 (b) and ROS (d). Fold changes of mRNA levels (a, c) are shown as 2^−∆∆Ct^ normalized to housekeeping gene *β*‐*ACTIN*. (f) Mean litter sizes of OoSirt1^+/+^ and OoSirt1^ΔEx4/ΔEx4^ females crossed with WT males. (g) Mean body weights of pups at 4 weeks of age. Oocyte and pup numbers are shown in parentheses. Scale bars = 20 µm. Data are shown as mean ± *SEM* (b, d, e–g) or as mean ± *SD* (a, c). Statistical analysis performed using two‐tailed Student's *t* test. ns denotes *p* > 0.05

To begin to investigate whether loss of oocyte‐Sirt1 impacted mitochondrial function and/or oxidative stress, we first analysed the expression of TFAM and MnSOD and found that mRNA levels of both were similar in OoSirt1^ΔEx4/ΔEx4^ and OoSirt1^+/+^ oocytes (Figure 1a). In keeping with normal TFAM levels, mitochondrial abundance measured using both TOM20 immunostaining and expression of the mitochondrial respiratory chain enzyme, CoxIV, were also indistinguishable between OoSirt1^ΔEx4/ΔEx4^ and OoSirt1^+/+^ oocytes (Figure 1b,c). Moreover, neither ROS nor ATP levels were affected by Sirt1 loss (Figure 1d,e), reaffirming intact antioxidant defences and mitochondrial respiration.

Entirely consistent with intact mitochondrial function and oocyte quality, young OoSirt1^ΔEx4/ΔEx4^ and OoSirt1^+/+^ females (2–3 months of age) produced similar numbers of pups after two mating rounds (Figure 1f). Moreover, pups born to OoSirt1^ΔEx4/ΔEx4^ mothers exhibited no overt physical abnormalities and were of comparable weights to pups from OoSirt1^+/+^ mothers (Figure 1g). Altogether, these data show that loss of Sirt1 does not impair oocyte developmental competence in vivo in young females.

### 2.3 Intact in vitro oocyte maturation and preimplantation embryo development whilst young

**FIGURE 2:**
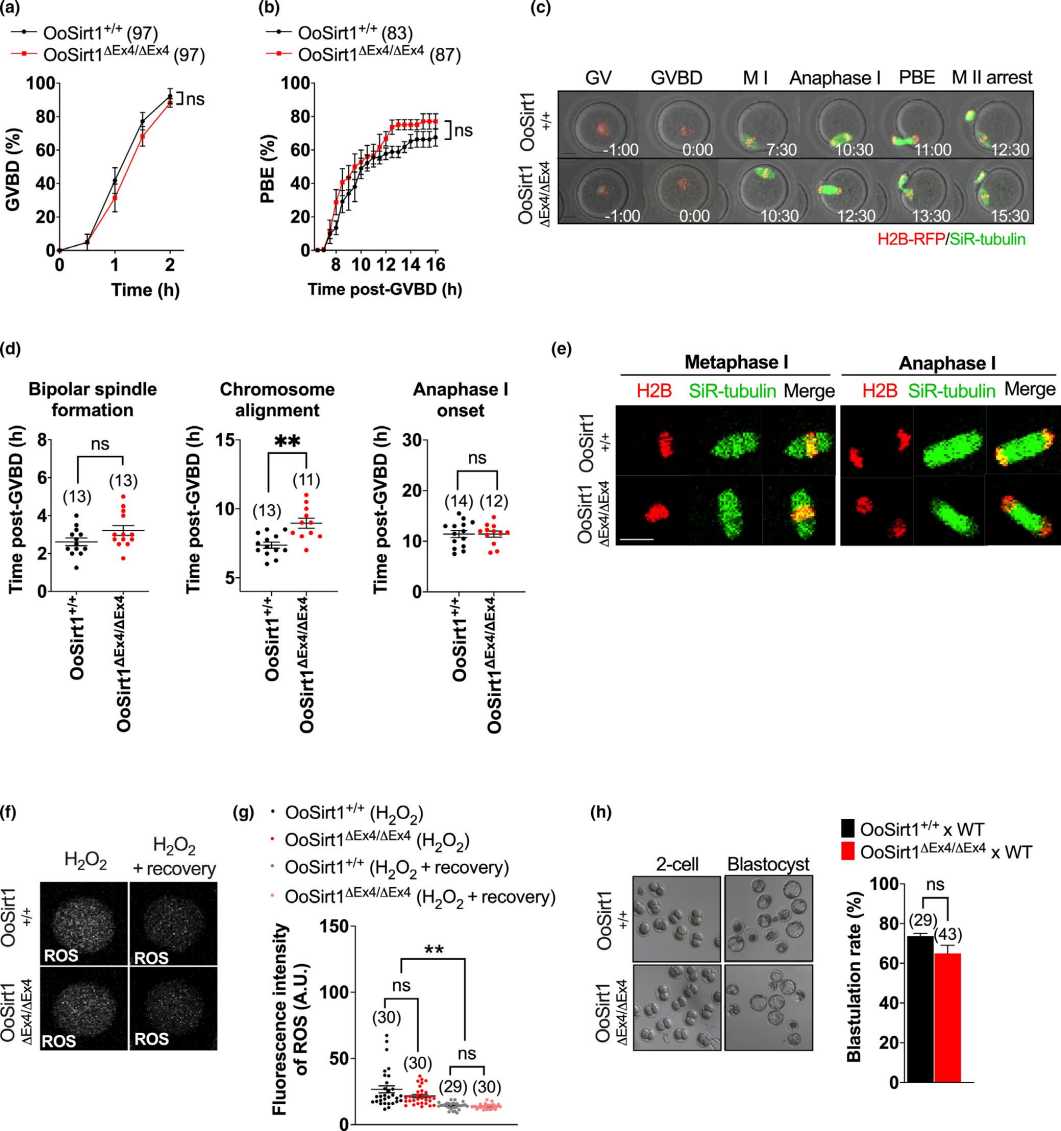
In vitro maturation, spindle assembly, chromosome alignment and segregation in oocytes and preimplantation development of embryos from young OoSirt1^+/+^ and OoSirt1^ΔEx4/ΔEx4^ females. Rates of (a) GVBD and (b) PBE. (c) Shown are panels comprised of selected brightfield and fluorescence frames from representative timelapse series of OoSirt1^ΔEx4/ΔEx4^ and OoSirt1^+/+^ oocytes. Time, h:min relative to GVBD. (d) Quantification of timing of bipolar spindle formation, chromosome alignment and anaphase I‐onset. (e) Shown are representative images of live OoSirt1^ΔEx4/ΔEx4^ and OoSirt1^+/+^ oocytes during metaphase I and anaphase I. (f) Shown are representative images of ROS fluorescence in oocytes immediately following peroxide treatment and after 90 min of recovery. (g) ROS quantification in peroxide treated OoSirt1^ΔEx4/ΔEx4^ and OoSirt1^+/+^ oocytes. (h) Blastulation rates of embryos derived from OoSirt1^ΔEx4/ΔEx4^ and OoSirt1^+/+^ females crossed with WT males. Shown are representative brightfield images of 2‐cell and blastocyst stage embryos. Oocyte and embryo numbers are shown in parentheses. Scale bar = 20 µm. Data are shown as mean ± SEM. Statistical analyses performed using two‐way Anova with Sidak's multiple comparisons test (a, b) or two‐tailed Student's *t* test (d, g, h). *p* values are represented as **p ≤ 0.01, ns denotes *p* > 0.05

In vitro treatment of denuded mouse oocytes with the Sirt1 inhibitor, Ex527, was previously found to impair ROS detoxification following exposure to peroxide‐induced oxidative stress (Di Emidio et al., 2014). This raised the possibility that surrounding follicular cells might help preserve the integrity of OoSirt1^ΔEx4/ΔEx4^ oocytes in vivo. If this were the case, then denudation would be detrimental to OoSirt1^ΔEx4/ΔEx4^ oocytes especially under the stressed conditions associated with in vitro culture (Kawamura et al., 2010). We tested this by undertaking >12 hr of timelapse confocal imaging of spindles and chromosomes in denuded oocytes. We found that entry into and exit from meiosis I (MI) marked by GV breakdown (GVBD) and first polar body extrusion (PBE), respectively, remained completely intact in OoSirt1^ΔEx4/ΔEx4^ oocytes (Figure 2a–c). Moreover, bipolar spindle assembly progressed normally, and although there was a modest delay in attaining metaphase I chromosome alignment, the overall ability to align chromosomes as well as the timing and integrity of anaphase I were unaffected (Figure 2d,e) (Video S1, S2).

To further test whether in vitro stressed conditions might expose a requirement for Sirt1, we treated young OoSirt1^ΔEx4/ΔEx4^ and OoSirt1^+/+^ oocytes with 20 µM hydrogen peroxide as before (Di Emidio et al., 2014). As expected, we found that ROS levels increased in both OoSirt1^ΔEx4/ΔEx4^ and OoSirt1^+/+^ oocytes immediately following peroxide treatment (Figure 2f,g). However, following 90 min of recovery, we found that both groups of oocytes were equally efficient at detoxifying ROS (Figure 2f,g).

Although loss of another sirtuin, Sirt3, has no overt in vivo effect on litter sizes under unstressed conditions (Iljas & Homer, 2020), preimplantation embryo development does become compromised under the stress of in vitro culture (Kawamura et al., 2010). We therefore wondered whether loss of Sirt1, whilst not affecting in vivo reproductive performance, might nevertheless impact in vitro embryo development. This was not the case, however, since blastocyst formation rates were around 60‐70% for embryos derived from both OoSirt1^ΔEx4/ΔEx4^ and OoSirt1^+/+^ oocytes (Figure 2h).

Thus, loss of oocyte‐Sirt1 in young females does not affect oocyte maturation, ROS detoxification or blastocyst development during in vitro culture.

### 2.4 Accelerated in vivo fertility decline following loss of Sirt1

Sirt1 is well known for its role during ageing in keeping with which, brain‐specific over‐expression of Sirt1 extends lifespan and delays ageing (Satoh et al., 2013). We therefore wondered whether loss of Sirt1 in oocytes, whilst having no effect in young oocytes, might become detrimental during ageing. To test this, we undertook an extended mating trial involving 16 OoSirt1^+/+^ females and 10 OoSirt1^ΔEx4/ΔEx4^ females commencing from 2 months of age through to 12 months when female mice exhibit clear features of reproductive ageing such as reduced oocyte numbers and litter sizes as well as increased oocyte‐derived aneuploidy (Pan, Ma, Zhu, & Schultz, 2008).

**FIGURE 3:**
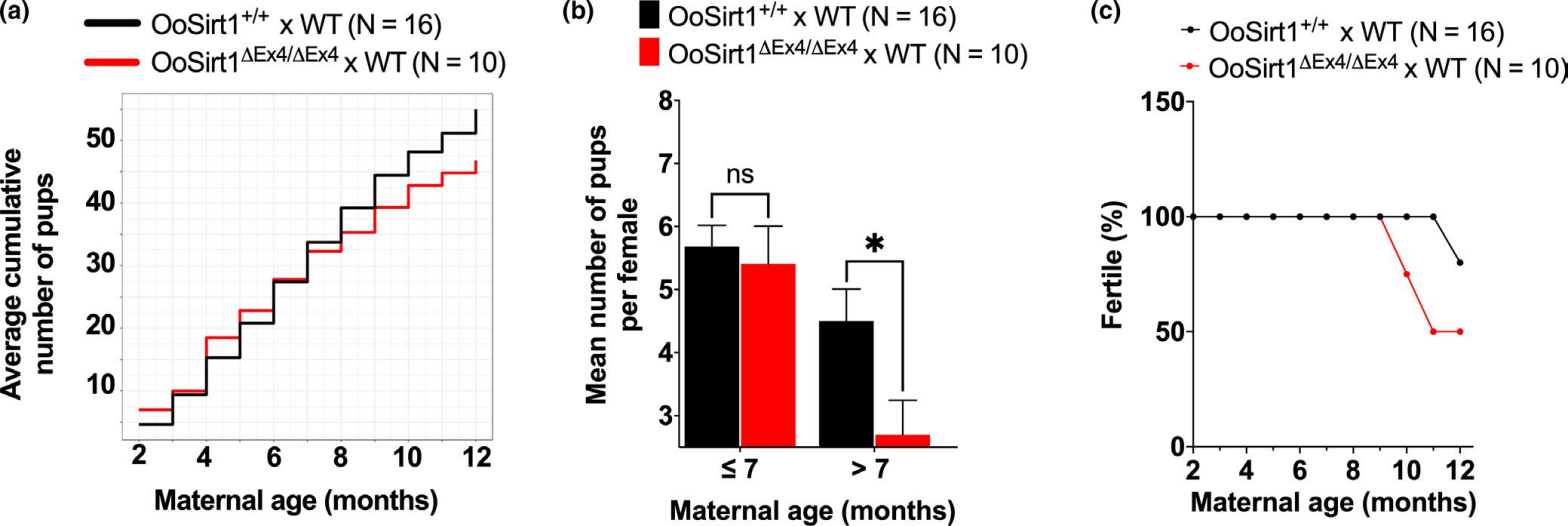
Fertility during female ageing. (a) Mean cumulative pup numbers for OoSirt1^ΔEx4/ΔEx4^ and OoSirt1^+/+^ females from 2 months to 12 months of age mated with young WT males. (b) Litter sizes of OoSirt1^ΔEx4/ΔEx4^ and OoSirt1^+/+^ females ≤7 months and >7 months. (c) Proportion of OoSirt1^ΔEx4/ΔEx4^ and OoSirt1^+/+^ females achieving live births (pregnancy rates) after mating with young WT males. Data are shown as mean ± *SEM*. Statistical analysis performed using two‐tailed Student's *t* test (b). *p* values are represented as *p ≤ 0.05, ns denotes *p* > 0.05. N, numbers of breeding pairs

In keeping with ageing effects, the numbers of pups produced when OoSirt1^+/+^ females were mated with wild‐type males declined as females got older (Figure 3a,b). Pup numbers produced when OoSirt1^ΔEx4/ΔEx4^ females were mated to wild‐type male studs also declined with age, but significantly, exhibited a more severe decline beyond 8 months of age when there was a mean of only 1 pup per female versus 4 pups per OoSirt1^+/+^ female (Figure 3a,b). Strikingly, we found that between 9 and 11 months of age, 50% of OoSirt1^ΔEx4/ΔEx^ females which had previously produced offspring became completely infertile despite evidence of continued vaginal plug formation (Figure 3c). In stark contrast, during this same period, 100% of OoSirt1^+/+^ females continued to have pups, albeit at reduced numbers (Figure 3c). Thus, loss of Sirt1 specifically in oocytes leads to a relatively sudden age‐related decline in fertility characterized by an overall reduction in mean numbers of pups as well as a complete loss of fertility in 50% of cases.

### 2.5 Age‐related impairment in oocyte quality in the absence of Sirt1

**FIGURE 4:**
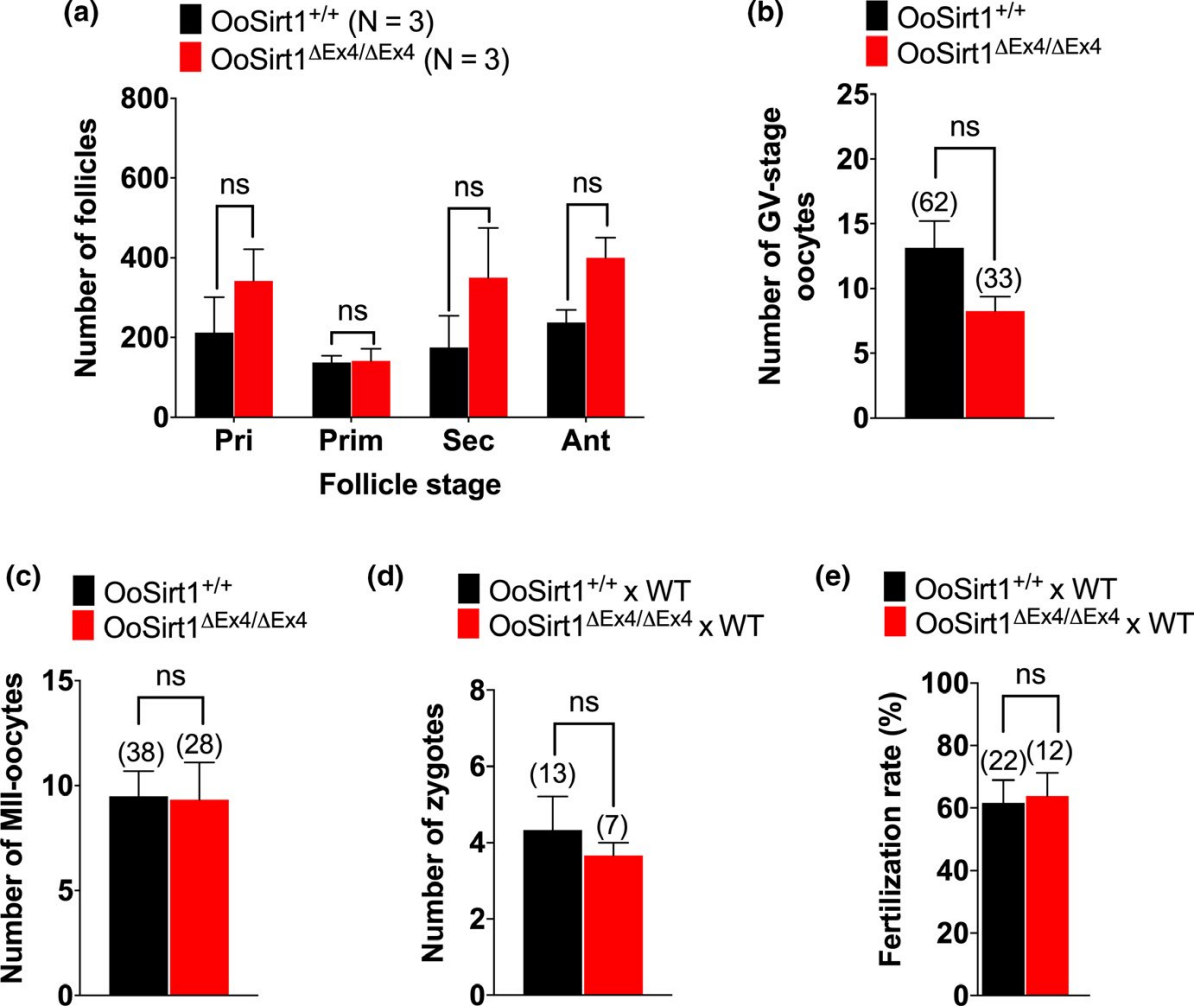
Oocyte numbers, in vivo maturation and fertilization in aged females. (a‐c) Quantification of (a) follicle numbers, (b) fully grown GV‐stage oocytes and (c) MII oocytes produced in vivo. (d, e) Numbers of zygotes (d) and fertilization rates (e) of OoSirt1^ΔEx4/ΔEx4^ and OoSirt1^+/+^ females crossed with WT males. Data are shown as mean ± *SEM*. Statistical analyses performed using either two‐way Anova with Sidak's multiple comparisons test (a) or two‐tailed Student's *t* test (b‐e). ns denotes *p* > 0.05. Oocyte and zygote numbers are shown in parantheses. N, numbers of mice

We wondered whether this accelerated age‐related fertility decline reflected a reduction in oocyte numbers and/or oocyte quality. To determine whether oocyte numbers were affected, we quantified follicle numbers in ovaries from 10‐ to 12‐month‐old OoSirt1^+/+^ and OoSirt1^ΔEx4/ΔEx4^ females and found that both groups had comparable numbers of all follicular stages (Figure 4a). In keeping with this, we obtained similar numbers of fully grown GV‐stage oocytes from both genotypes following hormonal stimulation (Figure 4b). Moreover, similar numbers of metaphase II‐arrested (MII) oocytes and zygotes were produced by aged OoSirt1^+/+^ and OoSirt1^ΔEx4/ΔEx4^ females and similar proportions of MII oocytes of both genotypes underwent fertilization in vivo (Figure 4c–e). Thus, reduced fertility in older OoSirt1^ΔEx4/ΔEx4^ females was not because of reduced oocyte numbers or due to compromised in vivo oocyte maturation or fertilization.

**FIGURE 5:**
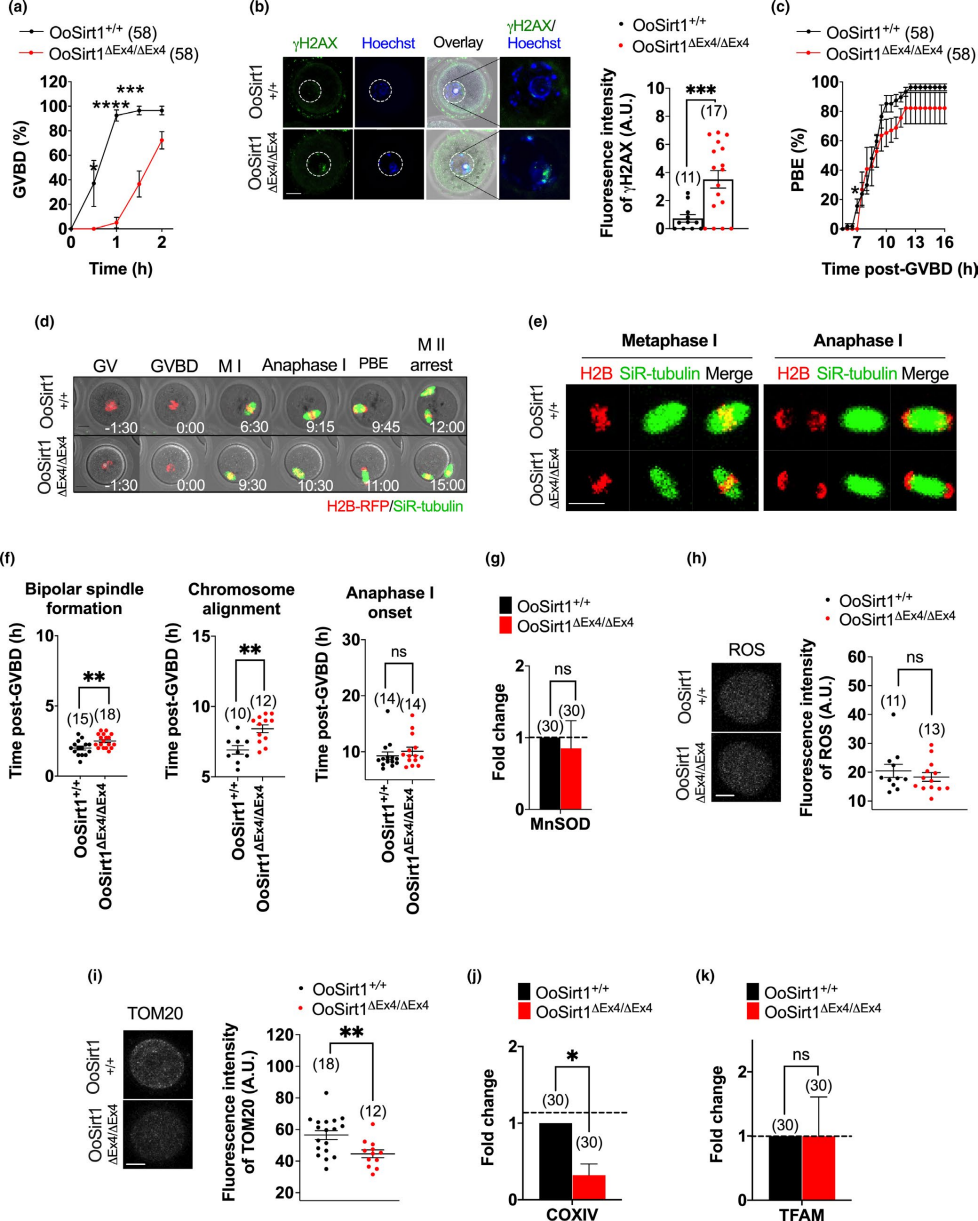
Ageing effects on oocyte quality. (a) GVBD rates. (b) Oocyte γH2AX levels. The white dashed line outlines the GV. (c) PBE rates. (d) Shown are panels comprised of selected brightfield and fluorescence frames from representative timelapse series of oocytes from aged mice. Time, h:min relative to GVBD. (e) Shown are representative images of live oocytes from aged mice during metaphase I and anaphase I. (f) Quantification of timing of bipolar spindle formation, chromosome alignment and anaphase I‐onset. (g–k) Levels of *MnSOD* mRNA (g), ROS (h), TOM20 (i), *COXIV* (j) and *TFAM* mRNA (k). Panels show images of representative oocytes stained for ROS (h) and TOM20 (i). Fold changes of mRNA levels are shown as 2^−∆∆Ct^ normalized to housekeeping gene *β*‐*ACTIN*. Scale bars = 20 µm. Data are shown as mean ± *SEM* (a–c, f, h, i) or shown as mean ± *SD* (g, j, k). Statistical analyses performed using either two‐way Anova with Sidak's multiple comparisons test (a, c) or two‐tailed Student's *t* test (b, f–k). *p* values are represented as *p ≤ 0.05, **p ≤ 0.01, ***p ≤ 0.001, ****p ≤ 0.0001, ns denotes *p* > 0.05. Oocyte numbers are shown in parentheses

The foregoing showed that OoSirt1^+/+^ and OoSirt1^ΔEx4/ΔEx4^ females produced similar numbers of zygotes but had reduced live births. From this, we infer that post‐fertilization development was compromised and, therefore, that loss of oocyte‐Sirt1 impairs oocyte developmental competence in aged females. Notably, however, impaired oocyte quality following loss of oocyte‐Sirt1 manifests relatively late in development, after oocyte maturation and fertilization. Although in vivo oocyte maturation remained intact, we reasoned that poor quality might compromise oocyte maturation under stressful laboratory culture conditions. We therefore studied in vitro maturation using long‐term timelapse imaging as before. Significantly, we found that whereas GVBD rates for OoSirt1^+/+^ oocytes attained ~90% by 1 hr following release from the reversible GVBD inhibitor, 3‐isobutyl‐1‐methylxanthine (IBMX), rates were less than 10% for OoSirt1^ΔEx4/ΔEx4^ oocytes (Figure 5a). OoSirt1^ΔEx4/ΔEx4^ GVBD rates did recover over the ensuing hour but remained lower than OoSirt1^+/+^ oocytes (Figure 5a). Since Sirt1 has been implicated in DNA repair (Wang et al., 2008) and longstanding DNA damage delays GVBD in mouse oocytes (Subramanian et al., 2020), we next investigated DNA damage levels. We immunostained oocytes for γH2AX, a known marker of DNA double‐strand breaks and found significantly higher γH2AX staining in OoSirt1^ΔEx4/ΔEx4^ oocytes (Figure 5b). Collectively, this suggests that loss of oocyte‐Sirt1 predisposes to increased DNA damage and is associated with delayed M‐phase entry.

Accompanying the reduction in GVBD was a non‐significant trend towards lower PBE rates for OoSirt1^ΔEx4/ΔEx4^ oocytes (Figure 5c). Using timelapse imaging, we did not observe any overt effects on spindle structure or chromosome alignment in aged OoSirt1^ΔEx4/ΔEx4^ oocytes (Figure 5d,e) (Video S3, S4) but did find that bipolar spindle assembly and chromosome alignment were significantly delayed albeit time of anaphase I onset was not affected (Figure 5f). Moreover, we never encountered lagging chromosomes during anaphase I suggesting that chromosome segregation remained intact in OoSirt1^ΔEx4/ΔEx4^ oocytes.

To interrogate oocyte quality further, we studied mitochondrial function and redox regulation. We found that levels of *MnSOD* mRNA and ROS were similar in aged OoSirt1^+/+^ and OoSirt1^ΔEx4/ΔEx4^ oocytes (Figure 5g,h) indicating no effect of Sirt1 loss on oxidative stress in oocytes. However, mitochondrial abundance measured using TOM20 immunostaining and CoxIV expression was significantly reduced following the loss of Sirt1 (Figure 5i,j). TFAM levels were not reduced, however (Figure 5k), suggesting that de‐regulation of another Sirt1‐dependent target was responsible for mitochondrial reduction.

Thus, there was evidence for compromised quality in aged OoSirt1^ΔEx4/ΔEx4^ oocytes manifested as increased DNA damage as well as impaired in vitro maturation, spindle assembly efficiency and mitochondrial abundance. This was not associated with impaired in vivo oocyte maturation or fertilization, however, suggesting that compromised oocyte quality following oocyte‐specific loss of Sirt1 impacted post‐fertilization development, ultimately culminating in infertility and reduced litter sizes.

### 2.6 Sirt1 loss impairs preimplantation embryo development via increased oxidative stress

Our data showed that oocyte‐specific Sirt1 loss had no overt effects at young ages but led to post‐fertilization defects with ageing. Because sirtuins have overlapping functions, we hypothesized that one or more sirtuins can cover for Sirt1 loss in young but not aged embryos. The decline in oocyte NAD^+^ levels we recently found to occur with ageing (Bertoldo et al., 2020) would compromise all sirtuins and could explain such a phenomenon. It is notable in this regard that preimplantation embryo development is extremely sensitive to reduced NAD^+^ levels brought about by inhibiting the NAD^+^ biosynthetic enzyme, Nicotinamide phophoribosyl‐transferase (Nampt), with the highly specific small molecule Nampt inhibitor, FK866 (Bertoldo et al., 2020).

**FIGURE 6:**
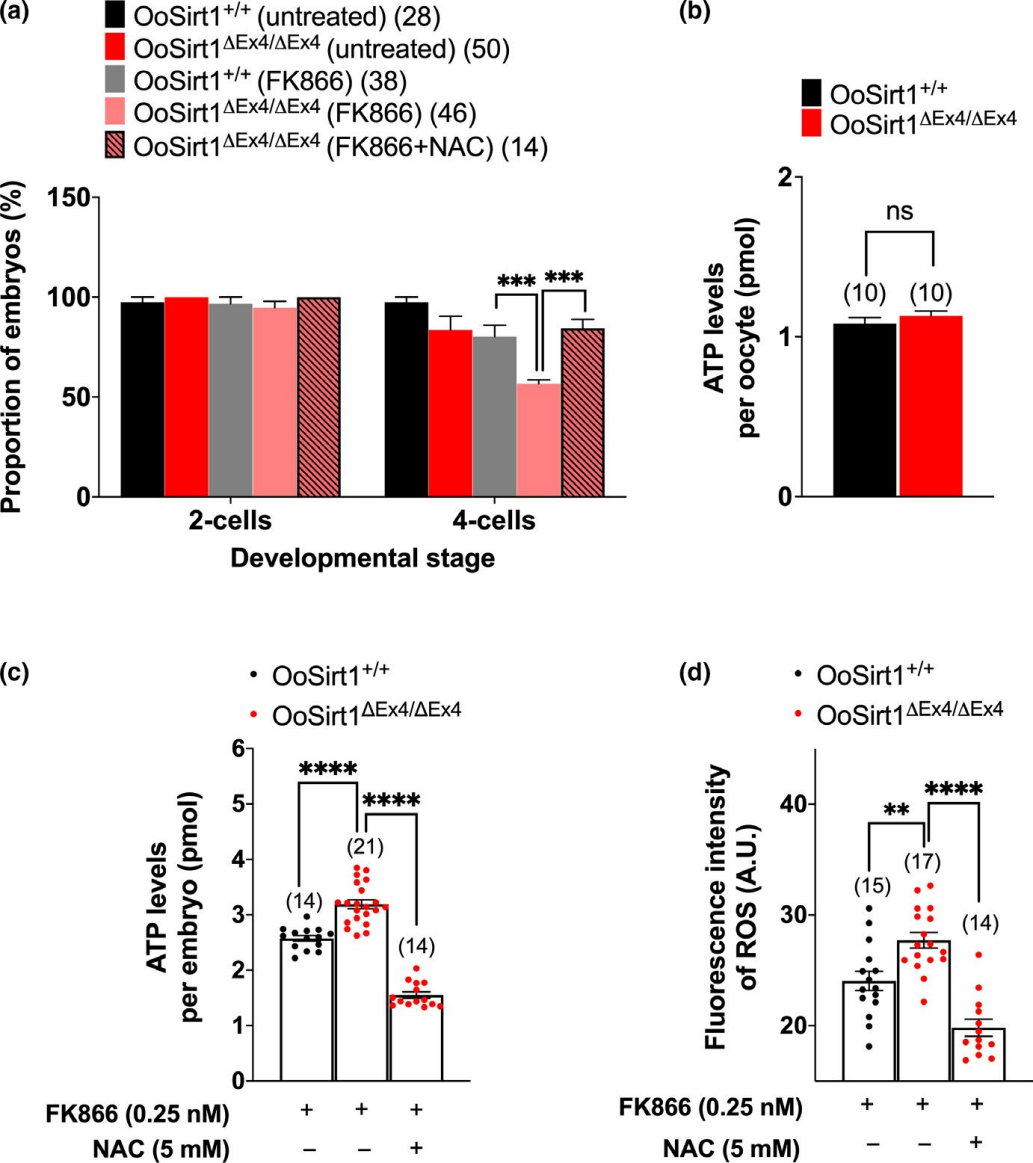
In vitro preimplantation development, oxidative stress and ATP levels of embryos following NAD^+^ depletion with FK866. (a) The proportion of zygotes, derived from females crossed with WT males, that reached 2‐cell and 4‐cell stages following treatment with FK866 and *N*‐acetyl cysteine (NAC). (b) Quantification of ATP levels in oocytes derived from aged females. (c–d) Quantification of ATP (c) and ROS (d) levels in 4‐cell embryos following treatment of zygotes with FK866 and NAC. Data are shown as mean ± *SEM*. Statistical analyses performed using either one‐way Anova with Tukey's multiple comparisons test (a, c, d) or two‐tailed Student's *t* test (b). *p* values are represented as **p ≤ 0.01, ***p ≤ 0.001, ****p ≤ 0.0001. Oocyte and embryo numbers are shown in parentheses

We therefore sought to investigate preimplantation embryos from aged females. Unfortunately, we could only obtain small numbers of in vivo‐generated zygotes from aged females (>10 months) and found that they developed very poorly under the added stress of in vitro conditions (data not shown) reminiscent of the sensitivity of Sirt3‐depleted embryos to in vitro culture (Kawamura et al., 2010). We therefore developed an in vitro model using FK866‐treated young embryos that would replicate reduced NAD^+^ levels brought about by ageing. Our model predicts that if reduced NAD^+^ erodes compensation by other sirtuins in aged embryos, then OoSirt1^ΔEx4/ΔEx4^ embryos would be particularly vulnerable to Nampt inhibition. Treatment of OoSirt1^ΔEx4/ΔEx4^ and OoSirt1^+/+^ zygotes with 0.25 nM FK866 did not affect development to the 2‐cell stage in either group (Figure 6a). Notably, however, and in line with our prediction, FK866 had no overt effects on subsequent progression to the 4‐cell stage in OoSirt1^+/+^ embryos but led to a marked reduction (~40%) in 4‐cell OoSirt1^ΔEx4/ΔEx4^ embryos (Figure 6a). Thus, these data suggest that in the presence of reduced NAD^+^ levels, cleavage stage divisions are disrupted in OoSirt1^ΔEx4/ΔEx4^ embryos perhaps explaining reduced litter sizes in aged OoSirt1^ΔEx4/ΔEx4^ females.

We reasoned that compromised embryonic cleavage might be due to reduced ATP availability since we found reduced mitochondrial levels in OoSirt1^ΔEx4/ΔEx4^ oocytes. Surprisingly, however, ATP levels were not reduced in either aged OoSirt1^ΔEx4/ΔEx4^ oocytes (Figure 6b) or FK866‐treated OoSirt1^ΔEx4/ΔEx4^ embryos (Figure 6c); indeed, FK866 led to higher ATP levels in OoSirt1^ΔEx4/ΔEx4^ embryos (Figure 6c). Strikingly, we found that ROS levels were elevated in FK866‐treated OoSirt1^ΔEx4/ΔEx4^ embryos (Figure 6d). Furthermore, reducing ROS levels by cotreatment with the antioxidant, NAC, completely rescued progression to the 4‐cell stage (Figure 6a,d). Interestingly, reduced ROS was associated with a reduction of ATP to normal levels (Figure 6c) suggesting that ATP production might somehow be responsive to ROS. Using Caspase‐3 immunostaining, we did not find any increased levels of apoptosis in OoSirt1^ΔEx4/ΔEx4^ embryos (Figure S3). Thus, increased oxidative stress is a major cause of compromised OoSirt1^ΔEx4/ΔEx4^ embryonic cleavage in the face of reduced NAD^+^ as occurs during ageing.

## 3 DISCUSSION

Here, we investigate the importance of oocyte‐Sirt1 for female fertility in vivo by deleting Sirt1 deacetylase activity specifically in growing oocytes. By deleting oocyte‐Sirt1 only, we eliminated confounding effects brought about by loss of Sirt1 from the soma. We find that at young ages, Sirt1 is completely dispensable in oocytes. Our results are in keeping with previous findings in young female Sirt1^−/−^ mice which produced normal numbers of oocytes when hormonally stimulated (Coussens et al., 2008) and young Sirt1^Y/Y^ females, which exhibited normal fertility (Seifert et al., 2012). An important proviso is that we only deleted Sirt1 exon 4 within the catalytic domain and not the entire gene. It therefore remains possible that the residual Sirt1 product exerts a function independent of Sirt1 catalytic activity that could ameliorate the phenotype in young animals. In line with distinct catalytic and non‐catalytic functions for Sirt1, the defects seen in Sirt1^Y/Y^ mice are markedly more subtle than in Sirt1^−/−^ mice (Seifert et al., 2012) and in an in vitro mouse ovary culture system, Sirt1 regulates oocyte transcription independent of its deacetylase activity (Zhang et al., 2019).

Notably, loss of oocyte‐Sirt1 exon 4 results in age‐accelerated female fertility decline that occurs quite suddenly and is not due to reduced oocyte numbers. Based on a 10‐month‐long breeding trial, we find that in the 2‐month interval from 9 to 11 months when 100% of OoSirt1^+/+^ females still produced live births, there was an abrupt decline in fertility in the absence of oocyte‐Sirt1 with 50% of OoSirt1^ΔEx4/ΔEx4^ females becoming infertile. In parallel, there was also a marked ~4‐fold reduction in mean pup numbers amongst OoSirt1^ΔEx4/ΔEx4^ females. It was somewhat surprising that loss of oocyte‐Sirt1 affected oocyte quality only and not numbers since increasing Sirt1 activity in vivo via calorie restriction, chemical activators or transgenic over‐expression all increase follicular reserve in aged females (Liu et al., 2013, 2015; Long et al., 2019; Zhou et al., 2014).

Although we found reduced mitochondrial abundance in aged OoSirt1^ΔEx4/ΔEx4^ females, there was no obvious reduction in ATP levels in either oocytes or embryos suggesting no overt impairment in OXPHOS. Instead, we found that increased oxidative stress in embryos was the critical downstream consequence of oocyte‐Sirt1 loss that derailed embryonic divisions. Interestingly, loss of oocyte‐Sirt1 did not affect basal oxidative stress in oocytes or the ability to counter peroxide‐induced stress. Thus, in the context of ageing, Sirt1 is reminiscent of a maternal‐effect gene (Li, Zheng, & Dean, 2010) insofar as it is produced by the oocyte but required later on during embryonic cleavage, in this instance, for combating oxidative stress. There are striking parallels between the ageing‐induced defects in preimplantation embryo development that likely occurs in vivo in OoSirt1^ΔEx4/ΔEx4^ females and the in vitro developmental compromise related to increased ROS observed in vitro in Sirt3‐deficient embryos (Kawamura et al., 2010).

This raises the intriguing question of why maternally derived Sirt1 is only required by embryos during ageing. Lack of adequate compensation by other NAD^+^‐dependent sirtuins offers a likely explanation. Two pre‐requisites for this hypothesis are firstly, that sirtuins have overlapping functions and are therefore able to cover for one another especially with respect to antioxidant defence and secondly, that changes brought about by ageing induce widespread compromise of sirtuin activity thereby impairing compensation. Regarding the former, it is well known that almost all sirtuins participate in oxidative stress management (Singh et al., 2018). Pertaining to the second pre‐requisite, a prominent consequence of ageing is a decline in NAD^+^ levels in oocytes (Bertoldo et al., 2020), which would impact all sirtuins. There are also reported declines in Sirt1, Sirt3 and Sirt6 levels in ovaries with ageing (Zhang, Fang, et al., 2016) and of Sirt1, Sirt2 and Sirt3 expression in oocytes with post‐ovulatory ageing (Zhang, Zhou, et al., 2016). We note that Sirt1, Sirt2, Sirt3 and Sirt6 all have functions in redox regulation (Singh et al., 2018). Consistent with this hypothesis, our ageing model created by reducing NAD^+^ levels via Nampt inhibition renders OoSirt1^ΔEx4/ΔEx4^ embryos uniquely vulnerable to increased oxidative stress and cleavage stage arrest.

Another interesting question pertains to why decompensation only occurs during embryonic divisions but not earlier during oocyte maturation. Highly significantly, it was previously reported that the expression of all seven sirtuins is very low during preimplantation embryonic stages compared with much higher levels in oocytes (Kawamura et al., 2010). Thus, preimplantation development typically occurs in a sirtuin‐poor environment leaving it exquisitely sensitive to any insult that further diminishes sirtuin activity. We propose that such insults occur during ageing and include reductions in expression of key sirtuins and importantly, reduced NAD^+^ availability.

Collectively, therefore, our data show that Sirt1 becomes critical for oocyte developmental competence during ageing likely because concurrent events compromise the ability of other sirtuins to suppress oxidative stress. Ultimately, in the absence of oocyte‐Sirt1, this leads to defects at the most vulnerable developmental stage, preimplantation embryos.

## 4 EXPERIMENTAL PROCEDURES

### 4.1 Animals and generating OoSirt1^ΔEx4/ΔEx4^ mice

Mice expressing Cre recombinase under the control of the *Zona pellucida 3* promoter (Zp3‐Cre mice) (Lewandoski et al., 1997) were purchased from the Jackson laboratory (Stock no. 003651; Bar Harbor, ME, USA). *Sirt1^f^* ^/^ *^f^* mice (Cheng et al., 2003) were a very kind gift from Dr. Lindsay Wu and Prof. David Sinclair (UNSW, Australia). The breeding strategy for generating OoSirt1^ΔEx4/ΔEx4^ mice is shown in Figure S1b. All animals were housed in a pathogen‐free environment in filter‐top cages at the animal facilities of the Herston Medical Research Centre and the University of Queensland Centre for Clinical Research. Mice were allowed *ad libitum* access to food and water and maintained in a facility with a 12 hr light/dark cycle at 22–24°C. All animal work complied with ethical regulations and were approved by the Animal Ethics Committee at the University of Queensland.

### 4.2 Oocyte collection, culture and microinjection

Oocytes were obtained from ovaries 44–48 hr following intra‐peritoneal injection of 5 international units (IU) of pregnant mare's serum gonadotropin (PMSG; Pacificvet) as described before (Gui & Homer, 2012, 2013; Homer, Gui, & Carroll, 2009; Wei, Greaney, Zhou, & Homer, 2018; Zhou, Hancock, Khanna, & Homer, 2019). Following humane euthanasia, ovaries were dissected and antral follicles were punctured using a 27 G needle (Terumo) under direct vision of a stereomicroscope (SMZ‐800N; Nikon) to release oocytes into pre‐warmed αMEM HEPES‐buffered medium supplemented with 50 μM 3‐isobutyl‐1‐methylxanthine (IBMX; Sigma), which prevents oocytes from undergoing GVBD. To study maturation, oocytes were allowed to resume meiosis by culturing them in micro‐drops of IBMX‐free M16 culture medium (Sigma) under embryo‐tested mineral oil (Sigma) at 37°C in an atmosphere of 5% CO_2_ and 5% O_2_. Generation of histone 2B (H2B)‐RFP cRNA (250 ng/µl) and microinjection into GV‐stage oocytes was performed as described previously (Subramanian et al., 2020; Wei et al., 2018; C. Zhou et al., 2019).

### 4.3 PCR genotyping

Genotyping was performed as described previously (Subramanian et al., 2020). Ear clippings and oocytes were lysed using Proteinase K (ThermoFisher) and incubated overnight at 55°C followed by inactivation at 85°C for 45 min. GoTaq Green Master mix (Promega) with forward and reverse primers (Biel et al., 2016) (Sigma; Table 1) were used for the PCR reaction. PCR products were separated on 2% agarose gels (Sigma) containing ethidium bromide (Omnipur; Merck). Images of gels were captured using a gel documentation system (Vilber Lourmat).

**TABLE 1:** Primer sequences for genotyping

Sequence (5′ → 3′)		
Gene	Forward	Reverse
*Cre*	AACCTGAAGATGTTCGCGAT	ACCGTCAGTACGTGAGATATC
*SIRT1^f/f^*	GCCCATTAAAGCAGTATGTG	CATGTAATCTCAACCTTGAG

### 4.4 Western blotting

Western blotting was performed as described previously (Gui & Homer, 2012, 2013; Homer et al., 2009; Subramanian et al., 2020; Wei et al., 2018; Zhou et al., 2019). Briefly, oocytes were lysed in LDS Sample Buffer (Invitrogen) followed by vigorous agitation to promote further lysis. Samples were boiled at 95°C for 5 min after addition of reducing agent (Invitrogen). Proteins were separated on 4‐12% Bis‐Tris gels (Invitrogen) and transferred to PVDF membranes (Merck). After overnight block, blots were probed with either anti‐Sirt1 (1:1000; Cell Signalling Technology) or anti‐actin (1:2000; Merck). Blots were washed and incubated for 1 hr in the appropriate HRP‐conjugated secondary antibodies (Bio‐Rad), exposed to ECL Pro solution (Perkin Elmer), and chemiluminescence signals were detected using ImageQuant LAS 500 (GE Healthcare Life Sciences).

### 4.5 Immunohistochemistry

Ovarian immunohistochemistry was performed as described previously (Subramanian et al., 2020). Ovaries were fixed in 4% paraformaldehyde (w/v) (Sigma), embedded in paraffin and serially sectioned at 5 μm and mounted on microscope slides (ThermoFisher). Antigen retrieval with 10 mM sodium citrate (Sigma) was used prior to incubation with primary anti‐mouse Vasa homolog (MVH) antibody (1:1000; Abcam). Endogenous peroxidase activity was quenched with 3% hydrogen peroxide (Sigma) followed by incubation with secondary HRP‐conjugated antibody and detected for HRP signals according to the manufacturer's instructions in the Dako EnVision Dual Link System‐HRP (DAB+) kit (Agilent Technologies). Sections were counterstained with Gill's Haematoxylin for 10 s. Every fifth section was photographed using a slide scanner (ScanScope XT; Leica Biosystems). Follicles at four different developmental stages (primordial, primary, secondary and antral) were classified as previously described (Myers, Britt, Wreford, Ebling, & Kerr, 2004). Follicles of every fifth section were counted as described before (Subramanian et al., 2020; Tilly, 2003).

### 4.6 Mating trials and preimplantation embryo culture

For mating trials, either OoSirt1^+/+^ or OoSirt1^ΔEx4/ΔEx4^ females were caged with young wild‐type (WT) males of proven fertility in a 1:1 ratio. Females were checked for vaginal plug formation as evidence of mating. Those identified to have plugs were then closely monitored for signs of pregnancy and thereafter monitored until delivery. At delivery, the numbers of pups per litter for each female were recorded. Females were maintained with males for a duration of 10 months.

To obtain mature oocytes or embryos, an additional injection of 5 IU human chorionic gonadotropin (hCG; MSD, Kenilworth, NJ, USA) was given 48 hr post‐PMSG injection. Unfertilized oocytes at metaphase II (MII) were collected from oviducts 22–24 hr post‐hCG injection. To obtain zygotes, either OoSirt1^+/+^ or OoSirt1^ΔEx4/ΔEx4^ females were caged in a 1:1 ratio with WT males immediately after hCG injection. Around 24 hr post‐hCG injection, females with a vaginal copulation plug were humanely euthanased. Oviducts were dissected, and zygotes were released into KSOM (Merck). Surrounding cumulus cells were removed using 300 µg/mL hyaluronidase (Sigma). After washing into hyaluronidase‐free KSOM, zygotes were cultured under mineral oil at 37°C, 5% CO_2_ and 5% O_2_. Zygotes were incubated in KSOM supplemented with 0.25 nM FK866 (NAMPT inhibitor; Apexbio) to replicate reduced NAD^+^ levels brought about by ageing. Oxidative stress was attenuated by cotreatment with *N*‐acetyl cysteine (NAC) (5 mM; 139476; Abcam). Media were changed daily. Embryo development was monitored daily for 4 days using an inverted brightfield microscope (Eclipse Ti U; Nikon). Fertilization rate was determined as the proportion of MII‐stage oocytes that contained visible pronuclei. Blastulation rate was calculated as the proportion of 2‐cell embryos reaching the blastocyst stage.

### 4.7 Immunofluorescence

Oocytes and embryos were fixed and stained as described previously (Gui & Homer, 2012, 2013; Homer et al., 2009; Subramanian et al., 2020). Briefly, oocytes and embryos were washed in PHEM buffer (pH = 6.9), pre‐permeabilized in 0.25% Triton X‐100 in PHEM and fixed in 3.7% paraformaldehyde in PHEM for 20 min. Oocytes and embryos were then blocked overnight at 4°C in PBS containing 3% BSA and 0.05% Tween‐20 before incubation with anti‐TOM20 (1:400; Santa‐Cruz) (Dalton & Carroll, 2013) or anti‐Caspase‐3 (1:400; Cell Signalling) for 1 hr at 37°C. Following washes, oocytes and embryos were incubated with Alexa Fluor 488‐conjugated secondary antibody (1:200, ThermoFisher) for 1 hr at 37°C, transferred to micro‐drops of PBS containing 0.5% BSA in glass‐bottom dishes (35 × 10 mm; MatTek Corporation) and imaged using confocal microscopy.

### 4.8 ROS and ATP measurements

To induce oxidative stress in vitro, GV‐stage oocytes were treated with 20 µM hydrogen peroxide for 30 min (Di Emidio et al., 2014). Oocytes were then incubated with 200 µM Oxidative Stress Detection Reagent (Abcam) for detection of ROS (Subramanian et al., 2020) either immediately or 90 min post‐peroxide treatment and then immediately imaged using confocal microscopy.

ATP content was determined using an ATP Bioluminescence Assay Kit (Sigma) as per manufacturer's protocol. Oocytes were lysed in 50 µl somatic cell ATP‐releasing agent. A volume of 100 µl of 1:5 diluted ATP assay mix was added to individual wells in an opaque 96‐well plate (ThermoFisher). Internal standards were prepared over the range of 0–1000 fmol/100 µl. 50 µl of either sample or standards were mixed with 100 µl ATP‐releasing agent and 50 µl ultrapure water to allow further ATP release. 100 µl of this mixture was transferred to the reaction wells, and emitted bioluminescence was immediately measured using the Spark 10 M Multimode Microplate Reader (Tecan Life Sciences). ATP levels in single oocytes were calculated based on the standard curve derived using internal standards.

### 4.9 Confocal microscopy

Confocal microscopy was performed using a Leica TCS SP8 microscope as described previously (Wei et al., 2018). Oocytes were imaged in micro‐drops of M16 medium in glass‐bottom dishes (MatTek), under mineral oil. For the entire duration of live‐cell imaging, oocytes were enclosed within a purpose‐built stage‐mounted incubation chamber designed to maintain conditions of 37°C and 5% CO_2_ in air. Automated image capture was driven by the Leica LAS X software.

To visualize microtubules, silicon rhodamine (SiR)‐tubulin dye (Lukinavicius et al., 2014) (Cytoskeleton Inc.) was added to media at a final concentration of 100 nM whilst H2B‐RFP was used to label chromosomes as before (Wei et al., 2018; C. Zhou et al., 2019). H2B‐RFP and SiR‐tubulin were imaged using the 561 nm and 633 nm laser lines, respectively, which were typically used at 0.5% and 3% power.

For quantifying TOM20, ROS and Caspase‐3 fluorescence, the 488 nm laser line was used and identical confocal settings were applied to all experimental groups. LAS X files were then imported into Image J software (NIH), and a maximum projection of the fluorescence channels was generated. The mean background‐corrected fluorescence intensity was then determined within a region encompassing the oocyte and normalized to maximum values. Post‐acquisition image processing was performed using Leica LAS X software, and images were assembled into panels using Microsoft PowerPoint. Panels were incorporated into figures using GraphPad Prism 8.2.1 (GraphPad Software).

### 4.10 Quantitative RT‐PCR

Total RNA was extracted from oocytes using the Arcturus PicoPure RNA isolation kit (Applied Biosystems) according to manufacture instructions. cDNA was synthesized using the Sensiscript reverse transcription kit (QIAGEN) and Oligo‐dT_12‐18_ primer (ThermoFisher) following the manufacturer's protocol. Quantiative real‐time RT‐PCR (qRT‐PCR) was performed using primers (Table 2) and PowerUpSYBR Green Master mix (Applied Biosystems) on the Quantstudio 3 Real‐Time PCR system (ThermoFisher). The 2^−∆∆CT^ (cycle threshold) method was used to calculate relative expression levels after normalization to β‐actin levels.

**TABLE 2:** Primer sequences for qRT‐PCR

Sequence (5′ → 3′)		
Gene	Forward	Reverse
*MnSOD*	GGTCGCTTACAGATTGCTGCC	TGCTCCCACACGTCAATCCC
*TFAM*	TCGCATCCCCTCGTCTATCAG	CCATGCTGGAAAAACACTTCGG
*COX IV*	GTCTTGGTCTTCCGGTTGCG	CTTGCCAATCAGGCTCAGCG
*β‐ACTIN*	ACCCTAAGGCCAACCGTGAA	GATGGCGTGAGGGAGAGCAT

### 4.11 Data presentation and statistical analysis

GraphPad Prism 8.2.1 (GraphPad) was used to calculate mean and standard error of the mean (SEM). qRT‐PCR data were processed in R (R Foundation for Statistical Computing, Vienna, Austria) to calculate fold change and standard deviation (SD). The statistical tests used for different data sets are detailed in the relevant figure legends. At least three biological replicates were performed for each experiment.

## CONFLICT OF INTEREST

HAH is a co‐founder, shareholder and advisor of Jumpstart Fertility Inc, which was founded to develop research into NAD+‐dependent pathways involved in female fertility.

## AUTHOR CONTRIBUTIONS

H. Homer designed research and wrote the paper. J. Iljas and Z. Wei performed research and analysed data.

## Supporting information

Figure S1

Figure S2

Figure S3

Video S1

Video S2

Video S3

Video S4

Supplementary Material

## Data Availability

The data that support the findings of this study are available from the corresponding author upon reasonable request.
