# Supplementary figures and images for: Sirt1 sustains female fertility by slowing age‐related decline in oocyte quality required for post‐fertilization embryo development
Source: Aging Cell. 2020 Jul 30;19(9):e13204. doi: 10.1111/acel.13204 (PMC7511857; doi:10.1111/acel.13204)

(a)

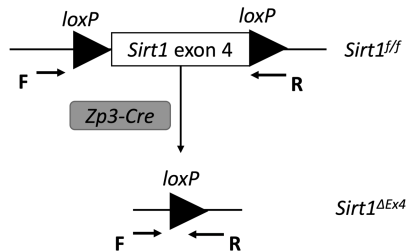

(b)

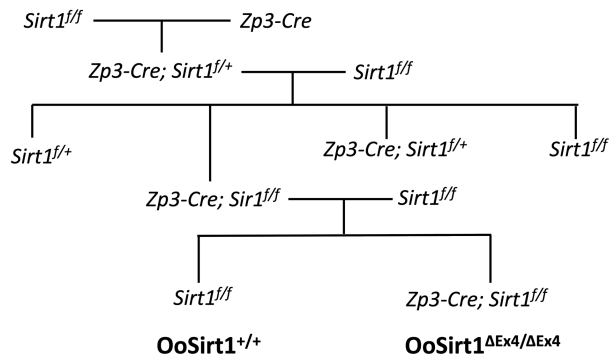

(c)

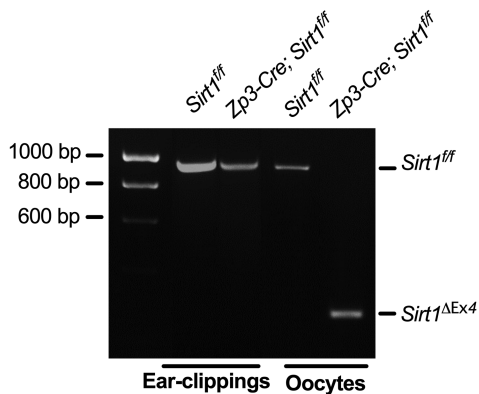

(d)

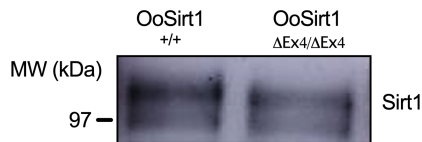

Supplement: Supplementary file 1 — Figure S1 [file ACEL-19-e13204-s001.pdf]

**(a)**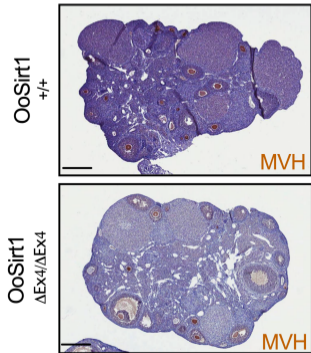**(b)**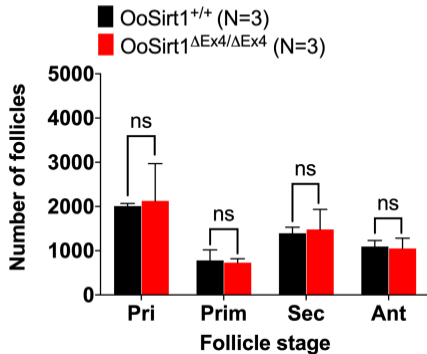**(c)**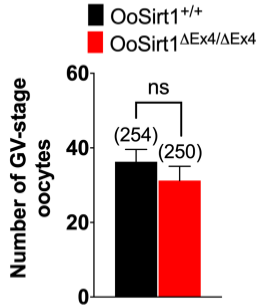

Supplement: Supplementary file 2 — Figure S2 [file ACEL-19-e13204-s002.pdf]
