# Supplementary material for: Sirt1 sustains female fertility by slowing age‐related decline in oocyte quality required for post‐fertilization embryo development
Source: Aging Cell. 2020 Jul 30;19(9):e13204. doi: 10.1111/acel.13204 (PMC7511857; doi:10.1111/acel.13204)
Supplement: Supplementary file 3 — Figure S3 [file ACEL-19-e13204-s003.pdf]

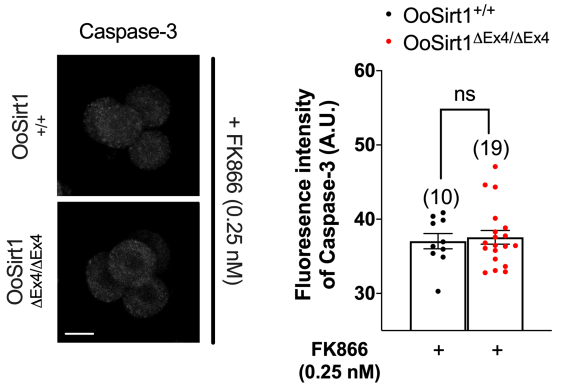

Caspase-3
OoSirt1 +/+
OoSirt1 ΔEx4/ΔEx4
+ FK866 (0.25 nM)
OoSirt1+/+
OoSirt1ΔEx4/ΔEx4
Fluoresence intensity of Caspase-3 (A.U.)
60
50
40
30
ns
(10)
(19)
FK866 (0.25 nM)
+
+
