# Supplementary material for: Sirt1 sustains female fertility by slowing age‐related decline in oocyte quality required for post‐fertilization embryo development
Source: Aging Cell. 2020 Jul 30;19(9):e13204. doi: 10.1111/acel.13204 (PMC7511857; doi:10.1111/acel.13204)
Supplement: Supplementary file 8 — Supplementary Material [file ACEL-19-e13204-s008.docx]

**Supporting Information listing**

**Supplemental Figure 1** Oocyte-specific deletion of exon 4 of *Sirt1*. (a) Schematic representation of *Zp3*-*Cre* mediated deletion of *Sirt1* exon 4 and creation of a truncated *Sirt1^ΔEx4^* allele in oocytes. F and R indicate the positions of the forward and reverse primers used for genotyping. (b) Schematic of breeding strategy for deleting exon 4 of *Sirt1* in oocytes. (c) Shown is a representative gel with the PCR products from ear-clippings and oocytes derived from *Sirt1^f/f^* and *Zp3-Cre; Sirt1^f/f^* females. Note that *Sirt1^ΔEx4^* is only found in oocytes expressing Cre recombinase (d) Shown is a representative Western blot of mouse GV-stage oocytes isolated from OoSirt1^+/+^ and OoSirt1^ΔEx4/ΔEx4^ mice (n = 32 oocytes per lane). Note that the Sirt1 product from OoSirt1^ΔEx4/ΔEx4^ mice migrates faster than the full-length product from OoSirt1^+/+^ mice.

**Supplemental Figure 2** Ovarian reserve in young OoSirt1^+/+^ and OoSirt1^ΔEx4/ΔEx4^ females. (a) Shown are representative images of ovarian sections stained with anti-MVH (brown), which specifically labels germ cells. (b) Quantification of primordial (Pri), primary (Prim), secondary (Sec) and antral (Ant) follicle numbers. (c) Quantification of numbers of fully-grown GV-stage oocytes obtained from hormonally primed females. Scale bar = 200 µm. Data are presented as the mean ± SEM. Statistical analyses performed using either Two-way Anova with Sidak’s multiple comparisons test (b) or Two-tailed Student’s *t* test (c). ns denotes *p* > 0.05. N, numbers of experimental mice. Oocyte numbers are shown in parentheses.

**Supplemental Figure 3** Apoptosis in 4-cell embryos following NAD^+^ depletion *in vitro*. Levels of Caspase-3 in 4-cell embryos following treatment of zygotes, derived from females mated with WT males, with FK866. Shown are representative images of Caspase-3 fluorescence in 4-cell embryos. Scale bar = 20 µm. Data are presented as the mean ± SEM. Statistical analysis performed using Two-tailed Student’s *t* test. ns denotes *p* > 0.05. Embryo numbers are shown in parentheses.

**Supplemental Movie S1.** Spindle assembly, chromosome alignment, anaphase I and PBE in an oocyte derived from a young OoSirt1^+/+^ female. Timelapse imaging of an OoSirt1^+/+^ oocyte expressing H2B-RFP (red) and stained with SiR-tubulin dye (green). Time scale is shown in hh:mm.

**Supplemental Movie S2.** Spindle assembly, chromosome alignment, anaphase I and PBE in an oocyte derived from a young OoSirt1^ΔEx4/ΔEx4^ female. Timelapse imaging of an OoSirt1^ΔEx4/ΔEx4^ oocyte expressing H2B-RFP (red) and stained with SiR-tubulin dye (green). Time scale is shown in hh:mm.

**Supplemental Movie S3.** Spindle assembly, chromosome alignment, anaphase I and PBE in an oocyte derived from an aged OoSirt1^+/+^ female. Timelapse imaging of an OoSirt1^+/+^ oocyte expressing H2B-RFP (red) and stained with SiR-tubulin dye (green). Time scale is shown in hh:mm.

**Supplemental Movie S4.** Spindle assembly, chromosome alignment, anaphase I and PBE in an oocyte derived from an aged OoSirt1^ΔEx4/ΔEx4^ female. Timelapse imaging of an OoSirt1^ΔEx4/ΔEx4^ oocyte expressing H2B-RFP (red) and stained with SiR-tubulin dye (green). Time scale is shown in hh:mm.
